# Catabolism of Fibromodulin in Developmental Rudiment and Pathologic Articular Cartilage Demonstrates Novel Roles for MMP-13 and ADAMTS-4 in C-terminal Processing of SLRPs

**DOI:** 10.3390/ijms20030579

**Published:** 2019-01-29

**Authors:** Cindy C Shu, Carl R Flannery, Christopher B Little, James Melrose

**Affiliations:** 1 Raymond Purves Research Laboratory, Institute of Bone & Joint Research, North Sydney Area Health Authority, Kolling Institute of Medical Research, University of Sydney, Royal North Shore Hospital, St. Leonards, NSW 2065, Australia; Cindy.shu@sydney.edu.au (C.C.S.); Christopher.little@sydney.edu.au (C.B.L.); 2 Bioventus LLC, 4721 Emperor Blvd., Suite 100, Durham, NC 27703, USA; carl.flannery@bioventusglobal.com; 3 Sydney Medical School, Northern, Royal North Shore Hospital, St. Leonards, NSW 2065, Australia; 4 Graduate School of Biomedical Engineering, University of New South Wales, Sydney 2033, Australia

**Keywords:** MMP-13, ADAMTS-4, FMOD, LUM, SLRPs, OA, PAMPs, DAMPs

## Abstract

Background: Cartilage regeneration requires a balance of anabolic and catabolic processes. Aim: To examine the susceptibility of fibromodulin (FMOD) and lumican (LUM) to degradation by MMP-13, ADAMTS-4 and ADAMTS-5, the three major degradative proteinases in articular cartilage, in cartilage development and in osteoarthritis (OA). Methods: Immunolocalization of FMOD and LUM in fetal foot and adult knee cartilages using an FMOD matrix metalloprotease (MMP)-13 neoepitope antibody (TsYG11) and C-terminal anti-FMOD (PR184) and anti-LUM (PR353) antibodies. The in vitro digestion of knee cartilage with MMP-13, A Disintegrin and Metalloprotease with Thrompospondin motifs (ADAMTS)-4 and ADAMTS-5, to assess whether FMOD and LUM fragments observed in Western blots of total knee replacement specimens could be generated. Normal ovine articular cartilage explants were cultured with interleukin (IL)-1 and Oncostatin-M (OSM) ± PGE3162689, a broad spectrum MMP inhibitor, to assess FMOD, LUM and collagen degradation. Results and Discussion: FMOD and LUM were immunolocalized in metatarsal and phalangeal fetal rudiment cartilages and growth plates. Antibody TsYG11 localized MMP-13-cleaved FMOD in the hypertrophic chondrocytes of the metatarsal growth plates. FMOD was more prominently localized in the superficial cartilage of normal and fibrillated zones in OA cartilage. TsYG11-positive FMOD was located deep in the cartilage samples. Ab TsYG11 identified FMOD fragmentation in Western blots of normal and fibrillated cartilage extracts and total knee replacement cartilage. The C-terminal anti-FMOD, Ab PR-184, failed to identify FMOD fragmentation due to C-terminal processing. The C-terminal LUM, Ab PR-353, identified three LUM fragments in OA cartilages. In vitro digestion of human knee cartilage with MMP-13, ADAMTS-4 and ADAMTS-5 generated FMOD fragments of 54, 45 and 32 kDa similar to in blots of OA cartilage; LUM was less susceptible to fragmentation. Ab PR-353 detected N-terminally processed LUM fragments of 39, 38 and 22 kDa in 65–80-year-old OA knee replacement cartilage. FMOD and LUM were differentially processed in MMP-13, ADAMTS-4 and ADAMTS-5 digestions. FMOD was susceptible to degradation by MMP-13, ADAMTS-4 and to a lesser extent by ADAMTS-5; however, LUM was not. MMP-13-cleaved FMOD in metatarsal and phalangeal fetal rudiment and growth plate cartilages suggested roles in skeletogenesis and OA pathogenesis. Explant cultures of ovine cartilage stimulated with IL-1/OSM ± PGE3162689 displayed GAG loss on day 5 due to ADAMTS activity. However, by day 12, the activation of proMMPs occurred as well as the degradation of FMOD and collagen. These changes were inhibited by PGE3162689, partly explaining the FMOD fragments seen in OA and the potential therapeutic utility of PGE3162689.

## 1. Introduction

The prevalence of knee osteoarthritis (OA) has doubled since the mid-twentieth century despite concerted efforts of the research and pharmaceutical industries over the last five decades to develop a therapeutic solution [1]. The global epidemic increase in body mass index (BMI) [2] may well have contributed to this higher incidence of OA [3,4] through a combined effect of joint overloading and adiposity-induced mild inflammation [5]. A Medline literature survey conducted in 2012 on OA and its associated medical costs based on three European, six North American and two Asian studies [6] showed that the annual cost of topical and oral non-steroidal anti-inflammatory drugs (NSAIDs) for the treatment of OA was US$57.38 million. Hip and knee replacement costs exceeded US$1087.43 million and arthroscopic OA surgery costs exceeded US$1.71 million. Indirect economic productivity loss due to OA cost over US$4.1 billion, community services expenditure amounted to US$5.25 million and social services were estimated to cost US$275 million [6]. A global OA study published in 2014, which examined the impact of knee and hip OA in 291 countries, confirmed these major socioeconomic impacts [3]. There remains a huge need to fully understand and treat OA and it is only through a better understanding of key disease processes that the research and pharmaceutical communities will be in a better position to finally resolve the therapeutic alleviation of this costly and currently intractable disease.

While OA is a disease of the entire joint, a pathognomonic feature is the progressive breakdown and loss of the articular cartilage leading to joint-organ failure and ultimately the need for replacement. In recent years, the enzymatic pathways responsible for degradation of the two key structural components of cartilage, aggrecan and type-2 collagen, have been defined and highlighted as therapeutic targets [7]. Rather than being a simple bi-molecular composite however, articular cartilage has a complex anisotropic 3-D structure and biochemical composition that enables it to fulfill its demanding biomechanical function in load-bearing joints. Articular cartilage contains several members of the small leucine-rich proteoglycan (SLRP) family with roles in extracellular matrix (ECM) assembly and cellular regulation [8,9,10], and has been shown in genetic studies to be critical to the maintenance of normal cartilage and joint function [11]. Fibromodulin (FMOD) and lumican (LUM) are horse-shoe shaped class II SLRPs [12] that are structurally homologous proteins sharing 47% identity in primary structure [13,14]. Like all class II SLRPs, FMOD and LUM contain 12 leucine-rich repeat domains (LRRs), which provide them with interactive properties with a number of extracellular matrix (ECM) proteins. Foremost in these interactions which organize tissue form and function is the interaction of FMOD and LUM with type I and type II fibrillar collagens. FMOD and LUM interact with the same binding sites on and regulate collagen fibrillogenesis in vitro [15]. FMOD-deficient mice have collagen fibrils of altered dimensions, and display OA-like features in their knee articular cartilage [16,17]. In addition, studies in knock-out mice indicate that LUM and FMOD reciprocally inhibit collagen interactions, through their binding sites on type I collagen [15]. The increased deposition of LUM in FMOD-deficient mice suggests that LUM binds to non-occupied FMOD-binding sites in collagen I [15].

FMOD and LUM are not redundant since they do not share functional equivalence in terms of how they organize ECM proteins. FMOD has N-linked glycosylation sites on Asn 127, 166, 201, 291 and 341, four of these sites are occupied by keratan sulfate (KS) at any one time. LUM contains N-linked KS chains located within the central LRR region at Asn 88, 127, 160, and 252. FMOD and LUM both contain N-terminal sulfated tyrosine residues; FMOD contains up to nine sulfated tyrosines and LUM contains two [18,19]. These are interactive with the heparin-binding proteins Fibroblast growth factor (FGF)-2, Thrombospondin (TSP)-I, MMP-13, the non-collagenous (NC)4 domain of collagen IX, and IL-10, and bind to collagen and promote fibril formation [20]. FMOD sequesters TGF-β in the ECM and controls its bio-availability, binds C1q and activates the complement system [21,22,23]. LUM differs from FMOD in terms of cell interactive properties, being an MMP-14 inhibitor and it impedes tumor cell migration and growth through its interaction with α2β1 integrin which conveys anti-angiogenic properties [24,25,26]. LUM also has novel roles in the innate immune response through interactions with Toll-like receptor-4 (TLR-4). LUM prevents or at least delays cleavage of collagen by MMP-13 in vitro [27].

We and others have shown extensive degradation of SLRPs including FMOD and LUM in OA articular cartilage [28,29]. In contrast to the proteolytic mechanisms for cartilage aggrecan and collagen degradation, the enzymes responsible for SLRP proteolysis in OA are less well-defined. In a study designed to examine the susceptibility of knee articular cartilage ECM components to MMP digestion, pieces of cartilage were used as substrates rather than soluble proteins, and proteomic sequencing was used to identify the unique peptides generated [30]. Peptides arising from biglycan (BGN), decorin (DCN), proline/arginine-rich end leucine-rich repeat protein (PRELP), FMOD and LUM were generated using MMP-2, -3, -8, -9, -12, -13, ADAMTS-4 and ADAMTS-5. ADAMTS-4 was the only MMP which degraded LUM, releasing two small peptides of low abundance. LUM was resistant to degradation by all the MMPs that were examined except MMP-12 which released three peptides. In comparison, FMOD was extensively degraded by all the MMPs tested with as many as 15 peptides generated by ADAMTS-4 and 18 peptides by MMP-12. The tyrosine sulfate-rich region of FMOD is also cleaved by MMP-13 with this event preceding enzymatic attack of the type II collagen fiber [31]. This work has clearly shown that FMOD and LUM have differential susceptibility to a number of enzymes known to be expressed in OA cartilage. The aim of the present study was to extend these previous observations using sequence-specific antibodies to examine FMOD and LUM degradation in OA and in the growth of articular cartilages, to further define the proteolytic pathways responsible for FMOD and LUM turnover in in health and disease.

## 2. Results

**Figure 1:**
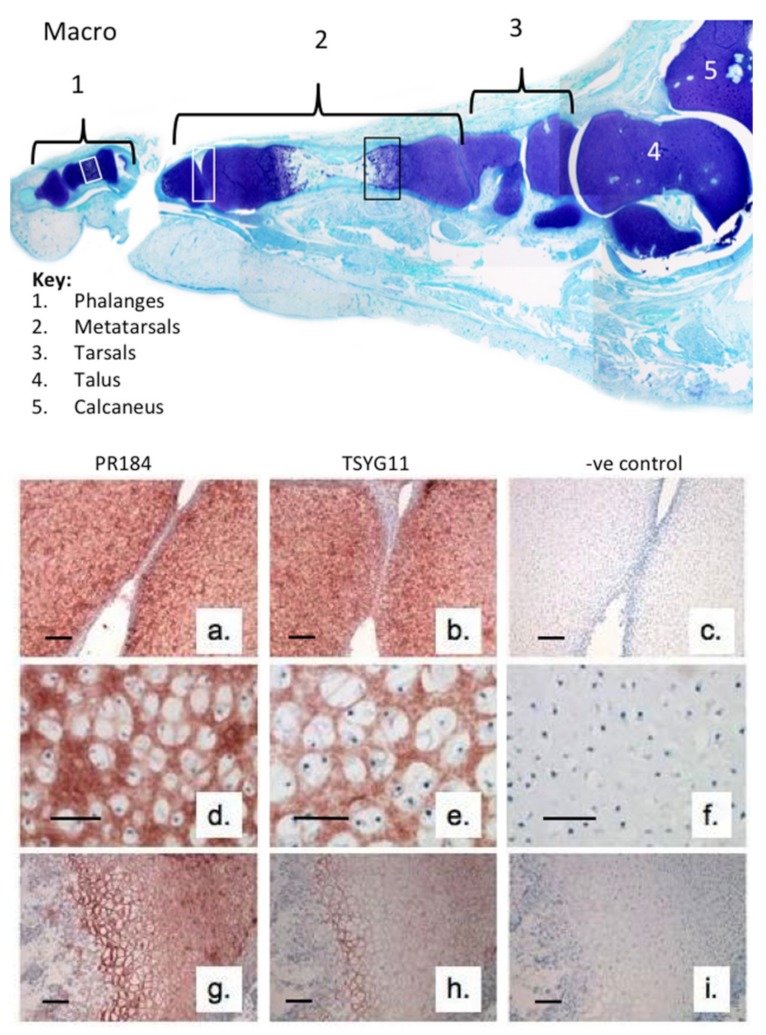
Macroscopic vertical section through a 14-week gestational age human fetal foot stained with toluidine blue-fast green (macroscopic views) to depict the cartilage rudiments and the areas used for immunolocalization (**a**–**i**). Immunolocalization of fibromodulin (FMOD) (pAb PR-184), scale bar = 100 µm (**a**,**d**,**g**) and MMP-13-cleaved FMOD (pAb TsYG11) (**b**,**e**,**h**) in human fetal (14-week gestational age) metatarsal and phalangeal rudiment cartilage (**a**,**b**; **d**,**e**) and metatarsal growth plates (**g**,**h**). Negative control sections are also depicted for the same cartilage areas (**c**,**f**,**i**). The immunolocalizations are representative of six sections from each sample zone taken from three feet samples.

We initially examined the immunolocalization of FMOD and MMP-13-cleaved FMOD in human developmental phalangeal and metatarsal cartilage rudiments and metatarsal growth plates (Figure 1). This demonstrated the prominent distribution of full-length FMOD in the foot rudiment cartilages associated with normal and hypertrophic chondrocytes (Figure 1a,d,g). MMP-13-cleaved FMOD displayed a similar distribution pattern in these tissues but was more prominently produced by the terminal hypertophic chondrocytes of the metatarsal growth plates (Figure 1h). This co-localization with the well-recognized region of MMP-13 activity in the growth plates [32] validated the TsYG11 antibody and the physiological role of FMOD proteolysis in rudiment cartilage development and bone formation.

**Figure 2:**
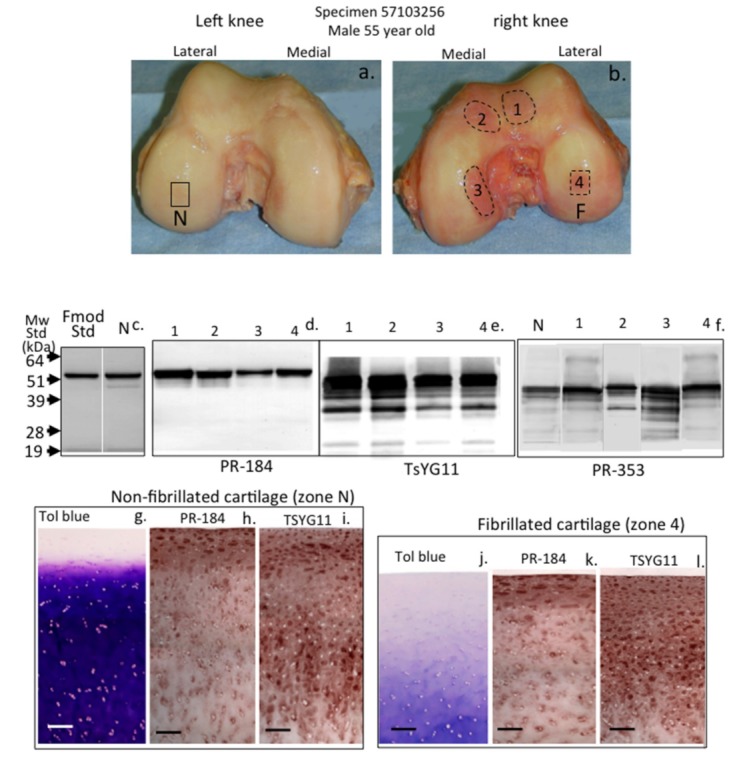
Macroscopic view of human knee femoral condyles from a 55-year-old male (**a**,**b**) with a normal (N) area devoid of surface fibrillation and four areas of fibrillated cartilage (1–4) which were extracted with 4 M GuHCl and analyzed by Western blotting (**c**,**d**,**e**,**f**) to identify FMOD (pAb PR-184) and MMP-13-cleaved FMOD (pAb TsYG11) and fragmented lumican (LUM) (pAb PR-353) in these regions. Toluidine blue stained proteoglycan distributions in non-fibrillated (**g**) and fibrillated cartilage (**j**) and FMOD (**h**,**k**) and MMP-13-cleaved FMOD (**i**,**l**) were also immunolocalized in full thickness cartilage sections from the non-fibrillated region (N) and fibrillated zone 4 (F). The blots shown are representative of three independent experiments. The immunohistology shown (**g**–**l**) was undertaken on four different tissue sections with qualitatively similar findings to those shown. Scale bar = 100 µm.

The visual macroscopic examination of human condyle articular cartilage identified areas of normal glistening macroscopically intact cartilage in the left knee of a 55-year-old male donor (Figure 2a). The right knee of this donor, however, contained extensive areas of surface roughened and fibrillated cartilage (Figure 2b). Four areas of degenerate cartilage and one normal cartilage region in the left knee were subsequently selected and these were extracted with 4 M GuHCl. The constituent FMOD and LUM species in these tissue extracts were identified using Western blotting and specific C-terminal antibodies to FMOD (PR-184) and LUM (PR-353) in normal (Figure 2c) and in fibrillated cartilage (Figure 2d,f). MMP-13-cleaved FMOD species were also identified on blots using pAb TsYG11 (Figure 2e). Very little fragmentation of FMOD was evident in the normal cartilage specimen (Figure 2c) or the fibrillated cartilage zones using pAb PR-184 (Figure 2d). Multiple MMP-13-cleaved FMOD fragments were, however, evident in all four fibrillated cartilage zones examined (Figure 2d). In contrast, fragmentation of LUM was evident in both the normal and fibrillated cartilage zones using pAb-353 (Figure 2e). There was a much more marked difference in LUM fragmentation between topographically-defined fibrillated cartilages, with degradation particularly prominent in zone 3 (Figure 2e). The histological examination of proteoglycan distribution, evident by toluidine blue-fast green staining in normal (Figure 2g) and fibrillated zone 4 (Figure 2j) cartilage regions in the lateral femoral condyles, displayed distinct differences. The cartilage thickness was significantly less in the zone 4 specimen and proteoglycan levels were severely depleted (compare Figure 2g with Figure 2j). Immunolocalization of FMOD and MMP-13-cleaved FMOD in the same cartilage regions demonstrated that full-length FMOD had a prominent distribution in the superficial cartilage with lower epitope levels deeper in the cartilages (Figure 2h,k), whereas MMP-13-cleaved FMOD was prominently distributed in both superficial and deep zones in normal and fibrillated cartilage (compare Figure 2i with Figure 2l). In general full-length and MMP-13-cleaved FMOD was distributed around the cells presumably associated with collagenous networks in the territorial matrix.

**Figure 3:**
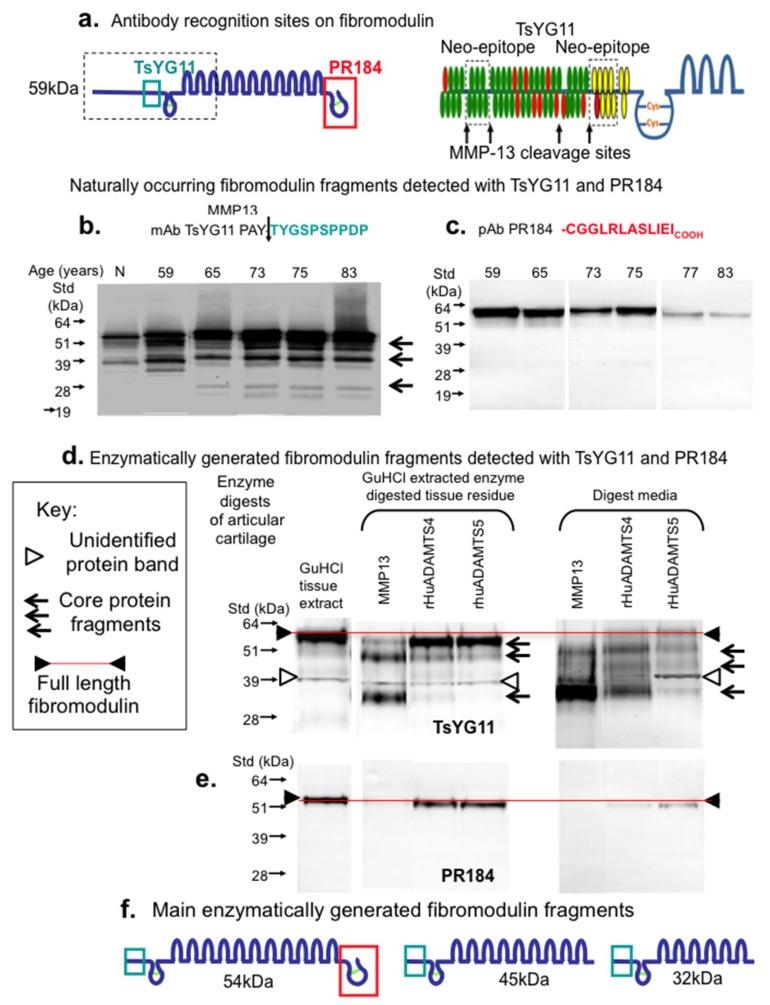
Schematic depiction of the epitopes identified by pAbs TsYG11 and PR-184 (**a**) and identification of naturally occurring FMOD fragments in OA knee cartilage from total knee replacement donors aged 59, 65, 75, 77 and 83 years of age using Western blotting and pAbs TsYG11 (**b**) and PR-184 (**c**) and FMOD species generated in vitro by enzymatic digestion using MMP-13, ADAMTS-4 and ADAMTS-5 detected using pAb TsYG11 (**d**) and pAb PR-184. Schematic depiction of the three major in vitro generated FMOD fragments. (**b**–**e**) were repeated three times and similar results were obtained. Schematic (**f**). depicts FMOD fragments identified.

Figure 3 schematically depicts the amino acid sequences and locations of areas of the FMOD core protein identified by the PR-184 and TsYG11 antibodies (Figure 3a). MMP-13 fragmented FMOD species were identified in extracts of normal non-degenerate and degenerate donor cartilages from total knee replacements donors aged 55, 58, 65, 73, 75 and 83 years of age (Figure 3b). Three prominent FMOD fragments were evident of 54, 45 and 32 kDa in size using the TsYG11 Ab (Figure 3b), while little if any fragmentation was detected using pAb PR-184 although band intensity was decreased in the older specimens (Figure 3c). Overnight enzymatic digestions of macroscopically normal articular cartilage from the femoral condyle of a 55-year-old male donor using MMP-13, ADAMTS-4 and ADAMTS-5 (Figure 3d) generated three FMOD fragments of similar sizes to those present in the OA cartilage extracts (Figure 3b). These fragments were most prominent in the MMP-13 digests and were both retained in the tissue (requiring release by GuHCl extraction) as well as being detected in the digestion media. ADAMTS-4 and ADAMTS-5 also generated FMOD fragments of a similar size and these were detected using pAb TsYG11 but to a lesser extent. The examination of the same digest sample blots with pAb PR-184 again failed to demonstrate FMOD fragmentation (Figure 3e). MMP-13 digests contained no detectable pAb PR-184-positive FMOD band, indicating that this enzymatic treatment degraded the C-terminal epitope. The putative three main enzymatically generated FMOD fragments based on size and differential antibody reactivity are presented schematically in Figure 3f.

**Figure 4:**
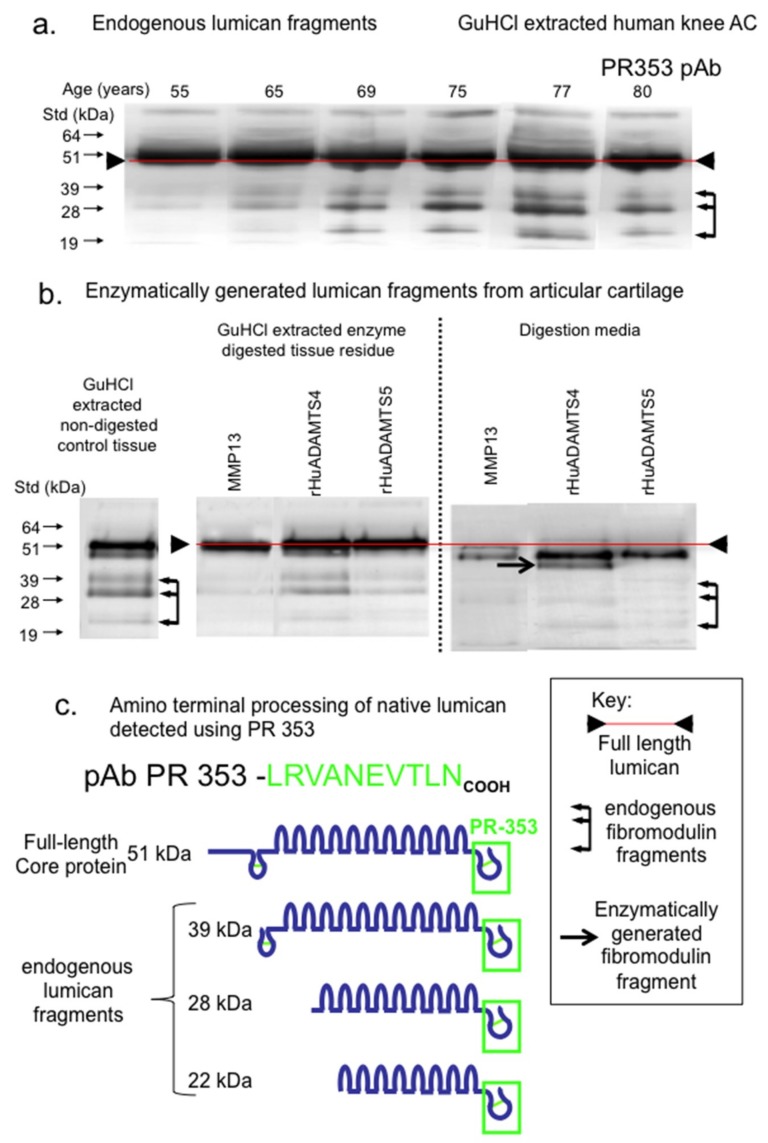
Composite figure depicting the naturally occurring LUM fragments in OA knee articular cartilage from total knee replacement donors aged 55, 65, 69, 75, 77 and 80 years of age (**a**) and released from macroscopically normal knee articular cartilage from the femoral condyle of a 55-year-old donor by enzymatic digestion with MMP-13, ADAMTS-4 or ADAMTS-5 (**b**). Schematic depiction of the major naturally occurring LUM fragments in OA knee articular cartilage, detected by pAb 353 on Western blots (**c**). Blots (**a**) and (**b**) were repeated twice and similar results were obtained in each case.

The examination of LUM fragmentation in knee cartilage extract samples by immunoblotting demonstrated three prominent LUM fragments using pAb PR-353 (Figure 4a). These were most prominent in the 69–80-year-old cartilage samples. Attempts to generate these LUM fragments by in vitro digestion of cartilage samples with MMP-13, ADAMTS-4 or ADAMTS-5 failed to generate the same LUM fragmentation pattern (Figure 4b). While similar LUM fragments were detected in GuHCl control extracts of cartilage, these fragments were not evident in the MMP-13 digests, suggesting that the fragments may have been further degraded by MMP-13. However, a full-length LUM band was still evident (Figure 4b), suggesting that the full-length protein may be more resistant due to its conformation and/or interaction with and protection by other ECM components. A predominant fragment slightly smaller than the full-length 51 kDa LUM was generated and released from cartilage by ADAMTS-4 digestion, along with minor levels of the other three aforementioned LUM fragments. ADAMTS-5 did not generate appreciable levels of LUM fragments (Figure 4b). The proposed LUM fragments observed are presented schematically in Figure 4c.

**Figure 5:**
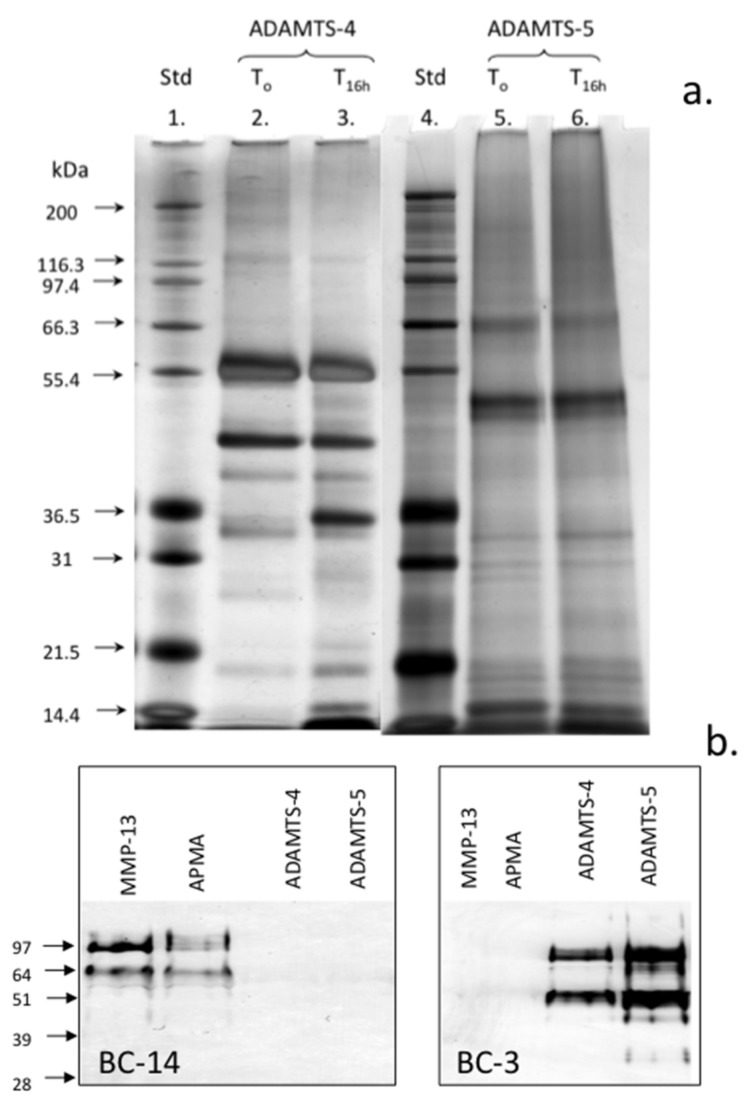
Assessment of the autocatalytic conversion of ADAMTS-4 and ADAMTS-5 in vitro. The silver-stained lithium dodecyl sulphate polyacrylamide gel electrophoresis (LDS PAGE) gel of the ADAMTS-4 and ADAMTS-5 species present following activation for 16 h. The samples of ADAMTS-4, -5 were incubated at 37 °C in MMP digestion buffer and the aliquots were removed after time zero and 16 h incubation at 37 °C and were then analyzed in pre-poured Bis-Tris 10% NuPAGE gels (0.5 µg/lane) electrophoresed in 3-(N-morpholino) propanesulfonic acid (MOPS) running buffer. The gels were stained with a Novex Silver Stain kit to visualize ADAMTS species (**a**). Western blots demonstrating the generation of the BC-14 MMP neoepitope by MMP-13 digestion of human articular cartilage and generation of the BC-3 aggrecanase neoepitope in aggrecan by the activated ADAMTS-4 and ADAMTS-5 preparations (**b**). Broad range protein molecular weight standards (Novex) were used in lanes 1 and 4 for size calibrations and SeeBlue-2 pre-stained standards for the Western blots. This experiment was repeated twice and similar results were obtained.

It is recognized that the substrate specificity of ADAMTS may be altered with C-terminal truncation of the enzymes [33] which is known to occur though autocatalysis during incubation [34,35]. The examination of the autocatalytic conversion of ADAMTS-4 and ADAMTS-5 demonstrated the generation of a number of smaller molecular weight forms in ADAMTS-4 but to a lesser extent in the ADAMTS-5 preparation (Figure 5a). These samples were nevertheless enzymatically active and cleavage of aggrecan in the cartilage samples used generated the typical BC-3 neoepitopes while MMP-13 or APMA did not (Figure 5b). MMP-13 (and APMA) did, however, generate the characteristic aggrecan MMP-cleavage neoepitopes identified by MAb BC-14 (Figure 5b).

**Figure 6:**
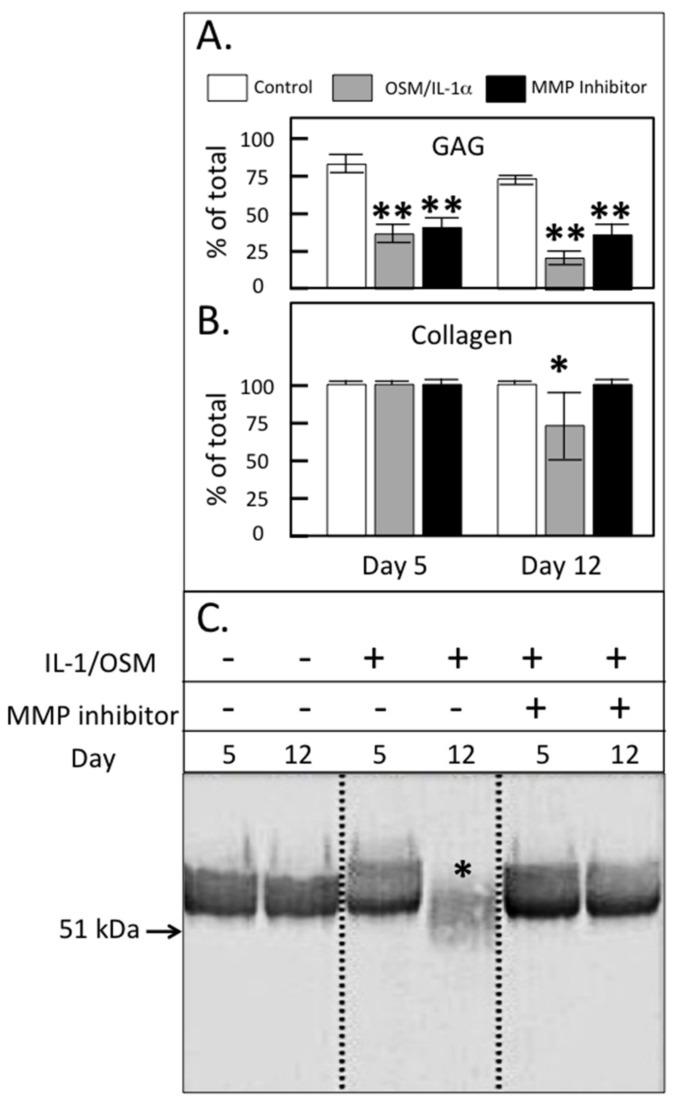
Glycosaminoglycan (GAG; **A**) and collagen content (**B**) of ovine articular cartilage explants after 5 or 12 days of culture in Dulbecco’s Modified Eagles Medium (DMEM) without (control) or with 5 ng/mL IL-1 and 50 ng/mL oncostatin M (IL-1/OSM) ± 300 nM MMP-inhibitor PGE3162689 (expressed as % of the total in explant plus media; mean ± standard deviation) (*n* = 6). Significant differences between control and treated cultures were defined as * *p* < 0.05, ** *p* < 0.01. (**C**) Western blot analysis of the FMOD core protein and the fragments in the cartilage extracts using PR-184, recognizing the C-terminus of FMOD. This blot was repeated three times.

The stimulation of the cultured ovine explants with IL-1α and oncostatin-M (OSM) was used to replicate chondrocyte-driven cartilage matrix degradation. As expected, IL-1/OSM resulted in a significant decline in cartilage sulfated glycosaminoglycan (GAG) levels on day 5–12 of culture, consistent with the stimulation of aggrecanolysis by this treatment (Figure 6a). Under the conditions used, inclusion of an MMP inhibitor failed to prevent this decline in GAG levels as expected with the concentration of PGE3162689 used (300 nM) where MMP-1, -2, -3, -7, -8, -9, -13 and -14 were inhibited but not ADAMTSs [36,37,38]. The cartilage collagen content (as measured by hydroxyproline) was unaffected by IL-1/OSM at day 5 but decreased in association with MMP activation on prolonged stimulation (Figure 6b, 12 days; collagen loss completely abrogated by inclusion of the MMP inhibitor). The immunoblotting of media samples from the explant cultures demonstrated a reduction in FMOD levels only on day 12 and the FMOD band was of a lower molecular weight than in the control cultures (Figure 6c). The inhibition of MMP activity by inclusion of PGE3162689 in the explant cultures prevented this proteolysis of FMOD, confirming that this was driven by MMPs and not ADAMTS.

## 3. Discussion

SLRPs have important roles to play in the organization of the cartilage ECM and functional roles in cartilage development remodeling and in the pathogenesis of OA [11]. In the present study, immunolocalization of FMOD in the developmental metatarsal and phalangeal rudiment cartilages and metatarsal growth plates of the foot demonstrated that FMOD was expressed by normal chondrocytes during cartilage development and by those chondrocytes undergoing hypertrophy in the growth plates. MMP-13-cleaved FMOD was prominent in the growth plates, which is consistent with MMP-13 as a marker of hypertrophy and with roles for FMOD and MMP-13 in endochondral ossification [39].

In the knee, OA is a progressive degenerative disorder affecting all joint tissues (articular cartilage, meniscus, synovium, subchondral bone, infrapatellar fat pad, ligaments) to variable degrees [40,41]. Historically, the degeneration of articular cartilage has been a major focus of studies on the etiopathogenesis of OA. However, with the appreciation of OA as a multifactorial global disorder and that degenerative changes in cartilage are affected by the synovium, subchondral bone, infra-patellar fat pad, meniscus, ligaments and tendons [40,41,42,43,44,45,46,47,48,49,50], there is now a greater appreciation of the contributions and feedback from and between all these joint tissues in the achievement of optimal knee functional properties [51,52,53,54]. A potential pathway exists whereby joint tissues may interact and contribute to the onset and progression of OA through the generation of a variety of damage-associated molecular pattern molecules (DAMPs) which act through multiple pathways [55]. DAMPs may reside inside the cell or are sequestered in the ECM in the healthy state [56,57] but their release from diseased/pathological tissues by proteases [58] makes them available to interact with pattern recognition receptors such as the Toll-like receptors (TLRs) and other non-immune cell-surface receptors which activate innate immune and inflammatory responses [59]. SLRPs can act as powerful DAMPs following their proteolytic release from the ECM, clustering different types of receptors to orchestrate a host of downstream signaling events [60,61,62].

During OA, active ADAMTS-4, ADAMTS-5 [63], MMP-2, MMP-3, MMP-13, and MMP-14 [30] could potentially release intact or fragmented forms of FMOD and LUM to act as DAMPs activating TLR2 and -4, initiating innate inflammation and pain pathways [58,62]. As shown by the present study, despite significant homology between FMOD and LUM, these SLRPs display differential susceptibilities to degradation by MMP-13, ADAMTS-4 and ADAMTS-5. FMOD is susceptible while LUM is resistant to MMP cleavage, which is potentially related to its identification as an MMP-inhibitor [64]. This inhibitory activity resides in a peptide named lumcorin, located in LRR-9 of LUM [65]. MT1-MMP has been shown to cleave LUM, abrogating the suppressive activity that it displays towards tumor cell colony formation [66]. The tyrosine-rich residues in the N-terminal region of FMOD are sulfated [18], bind collagen enhancing fibril formation [20], and heparin binding bioactive factors [20] but are cleaved by MMP-13 [67]. FMOD in articular cartilage, and meniscus is extensively fragmented in human [29] and animal models [68] of OA. In the present study, intact FMOD was present at a higher density in the superficial region than the interterritorial matrix of knee articular cartilage as previously reported [69]. While similarly present in the superficial zone, MMP-13-cleaved FMOD was also prominent in the deep cartilage regions consistent with cleavage by chondrocyte rather synovial-derived enzymes. While FMOD fragmentation has been reported in RA and OA articular cartilage [28,70] and with ageing [71], we have recently found minimal fragmentation compared with other SLRPs in OA knee menisci and knee and hip articular cartilage when a C-terminal antibody was used for FMOD detection [72]. The present study shows that C-terminal processing of FMOD may have resulted in an inability to detect the processed forms of FMOD using Ab PR 184 in this earlier study.

In cartilage degradation in vitro, FMOD is degraded in bovine nasal cartilage explant cultures stimulated with IL-1 [67,73]. Cleavage in the N-terminal extension of FMOD resulted in the release of all but one of its sulfated tyrosine residues [67]. In-vitro experiments demonstrated that MMP-13 but not MMP-2, -3, -8, or -9, selectively cleaved at this N-terminal site, but only when FMOD was bound to type II collagen and not when in free solution [67]. This work resulted in the development of the MMP-13 neoepitope antibody (TsYG11) to the MMP-13 cleavage sequence in bovine FMOD between residues 63 and 64 at PAY^63^↓A^64^YG [67] that we used this antibody in the current work.

The digestion of the macroscopically intact articular cartilage with MMP-13 generated three TsYG11-positive FMOD fragments of Mw 54, 45 and 32 kDa. Previous studies have demonstrated the cleavage of soluble bovine FMOD by ADAMTS-4 and ADAMTS-5 [33,74]. In the current study, we confirmed that ADAMTS-4 and ADAMTS-5 also generated FMOD fragments of 54 and 45 kDa through cleavage at the MMP-13 sensitive sites (TsYG11-positive). While resistant to MMPs, the examination of LUM fragmentation in ADAMTS-4 and ADAMTS-5 digested cartilage samples identified two prominent fragments of 48 and 45 kDa. These findings are in agreement with an earlier study where cartilage pieces were digested with MMP-2, -3, -8, -9, -12, -13 and ADAMTS-4 and ADAMTS-5 [30].

A single MMP-13 cleavage site has been identified in bovine FMOD, and we show that ADAMTS-4 and ADAMTS-5 also cleave at this site, generating a reduction in size of approximately 5–10 kDa [33,67,74]. A similar fragment was also identified in OA human knee cartilage. These TsYG11-positive fragments most likely arose from proteolysis at the known MMP-13 cleavage site as well as one of several as yet unidentified cleavage sites in the C-terminus and LRRs. In the present study, recombinant MMP-13 and ADAMTS-4 generated a 37 kDa fragment also observed in OA tissues. ADAMTS-4 and ADAMTS-5 also cleave FMOD at the same Tyr-Ala bond as MMP-13, with ADAMTS-5 potentially being more active against FMOD in solution-phase digests [74,75]. Our results demonstrated that ADAMTS-4 degraded FMOD in cartilage, despite ADAMTS-5 showing superior aggrecanolytic activity in vitro. While generated by incubation with recombinant enzymes, stimulation of cartilage explants with IL-1/OSM suggested that chondrocyte-derived MMP-13 and not ADAMTS plays a greater role. Thus, despite the potential for ADAMTS enzymes, and ADAMTS-4 in particular, to cleave FMOD, MMP-13 likely plays a more significant role. Thus, our in vitro data support previous reports [67,76] that cleavage of FMOD occurs after the major breakdown of aggrecan by ADAMTS but before significant MMP-driven collagenolysis has occurred, suggesting that removal of FMOD from the collagen fibril may be a prerequisite for collagen proteolysis. The susceptibility to ADAMTS proteolysis of LUM, which has similar collagen binding, may also contribute to sequential cartilage ECM degradation in OA.

The identification of the specific fragments of FMOD that are released from cartilage may provide useful biomarkers to monitor the progression of cartilage degradation from early stage aggrecan loss to later collagenolysis and to identify therapeutic molecular targets. The differential susceptibility of FMOD and LUM to enzymatic degradation by MMPs observed in the present study was a surprising finding but in keeping with a recent study which demonstrated that LUM was an MMP-14 inhibitor [64]. MMPs are generally considered the physiological modulators of connective tissue composition both in tissue development and in pathological degradation. However, LUM appears to be susceptible to either an MMP other than that which was examined in this study or the cited studies or another class of proteinase. A related SLRP member mimecan/osteoglycin is degraded by BMP-1 which converts it to a form with better interactive properties with collagen fibrils [77]. A similar proteinase may also be responsible for the LUM fragmentation observed in pathological knee cartilage samples.

## 4. Materials and Methods

### 4.1. Consumables

All electrophoresis consumables, pre-poured gels, running buffer concentrates, blotting membranes and pre-stained protein molecular weight standards were obtained from Invitrogen, Mount Vic, Australia. Blotting consumables, NBT/BCIP substrates, alkaline phosphatase labeled secondary antibodies and development buffers were obtained from Bio-rad Laboratories Pty Ltd, Gladesville, NSW 2111, Australia. Chondroitinase ABC (*Proteus vulgaris*), keratanase-I (*Pseudomonas* sp.) and guanidine hydrochloride (GuHCl) were obtained from Sigma-Aldrich Australia, Castle Hill, NSW, Australia. *N*-glycanase (peptide-*N*-glycosidase F) from *E. coli* was obtained from Genzyme, N. Ryde, NSW, Australia. Recombinant human pro-MMP-13 was a generous gift from Professor Gillian Murphy, University of Nottingham, Nottingham, UK Recombinant human ADAMTS-4 and ADAMTS-5 were kindly supplied by Dr Carl Flannery, Wyeth, Cambridge, Mass, USA. Menzel and Glaser SuperFrost ultraPlus, positively-charged microscope slides were obtained from Fisher Scientific, Braunschweig, GmbH. NovaRED peroxidase substrate was obtained from Vector Laboratories (Burlingame, CA, USA). Biotinylated anti-rabbit IgG secondary antibody, avidin HRP conjugate and non-protein block were obtained from Dako, Botany, NSW, Australia. Fetal calf serum was obtained from Trace Biosciences Pty. Ltd., Castle Hill, NSW, Australia.

### 4.2. Tissues

This study was approved by the Human Research Ethics Committee of the Royal North Shore Hospital; all discarded tissues at the time of knee-joint replacement surgery were obtained with informed consent. This work was covered by two ethics covers (i) NSLHD Protocol 0404-103M (Oct 2004–2008): J Melrose, C Little, D Sonnabend—“Pathobiology of the small leucine rich repeat proteoglycans in cartilage, intervertebral disc and tendon degeneration” for use of discarded human tissues from surgery and by NSLHD Protocol 0308-163M (Sept 2004–2009): J Melrose, C Jackson, J Whitelock—“The role of perlecan in cartilage development” which covered use of human foetal joint tissues.

Cartilage from a total of 25 total knee replacements was collected for this study. Four non-arthritic knees were obtained from The International Institute of Advancement in Medicine (IIAM), Jessup, PA, USA; a division of the Musculoskeletal Foundation. Cartilage from these normal joints was harvested from regions with a normal smooth glistening surface (N) and regions with a dull appearance indicative of surface fibrillation. Human fetal feet (12–14 week gestational age) were obtained at the time of termination of pregnancy with ethical permission. Human fetal feet (*n* = 4) were selected as a tissue to study since we were interested in examining the growth plates as well as chondrocytes in the main rudiment cartilage and these specimens conveniently contain around 30 articulating joints in the one specimen. The growth plate is a well known area of MMP activation and growth plate chondrocyte hypertrophy is associated with MMP-13 activity. Thus, these tissues can be used to demonstrate proof of principle and the authenticity of the antibody preparations. Adult human knees were used since these provide multiple cartilage samples for analysis from morphologically normal and surface abraded OA cartilage regions. Sheep stifle joint control tissues were obtained from related projects in our laboratory for comparative in vitro analyses with IL-1/OSM.

### 4.3. Antibodies

Affinity purified rabbit polyclonal antibodies to the C-terminal peptide sequence LRLASLIEI of human FMOD (Ab PR-184) were used as described earlier [72,78]. The anti-C-terminal LUM antibody used in this study was an affinity purified rabbit polyclonal antibody raised against the peptide sequence H-CGGLRVANEVTLN-OH, which comprises an amino terminal cysteine residue used for conjugation to ovalbumin for antibody production, two spacer glycine residues, and the carboxyl terminal 10 amino acids of human, bovine and chick LUM. The anti-LUM antibodies were purified by affinity chromatography using the same immunization peptide as ligand. A rabbit polyclonal antibody (TsYG11) to the ten amino acid linear sequence TYGSPSPPDP C-terminal to the putative MMP-13 cleavage site in human FMOD was generated to examine FMOD core protein fragmentation by Associate Prof Patrik Onnerfjord and Prof Dick Heinegard (deceased) University of Lund, Sweden. Monoclonal antibodies to neoepitope sequences generated by cleavage of aggrecan by ADAMTS (ARGS…; antibody BC-3) or MMPs (FFGV…; BC-14) were provided as hybridoma culture supernatant by Professor Bruce Caterson and Dr Clare Hughes (Cardiff University) and used to confirm that ADAMTS-4 and ADAMTS-5 were enzymatically active prior to digestion of cartilage.

### 4.4. Extraction of Tissues

The tissues were cut into small pieces using scalpels and extracted with 10 volumes of 4 M guanidine hydrochloride (GuHCl) and 0.5 M sodium acetate, pH 5.8, containing 10 mM EDTA, 20 mM benzamidine and 50 mM 6-aminohexanoic acid using end-over-end mixing for 48 h at 4 °C. The tissue residues were separated from the extracts by centrifugation and discarded. Tissue extracts were dialyzed against 3 changes of milliQ water and freeze dried.

### 4.5. Chondroitinase ABC, Keratanase-I and N-Glycanase Digestion of Tissue Extracts

The freeze dried tissue extracts were re-dissolved (2 mg dry wt/mL) overnight in 100 mM Tris 0.03 M acetate buffer, pH 6.5, at 4 °C with constant end-over-end mixing and the aliquots (0.5 mL) were digested with chondroitinase ABC (0.1 U) and keratanase-I (0.05 U) overnight at 37 °C. Selected tissue extracts were also digested with N-glycanase (PNGase F, Prozyme laboratories, was obtained through Agilent Technologies, Mulgrave, Vic, Australia Briefly, chondroitinase ABC and keratanase-I digested samples were dialyzed and freeze dried, then re-dissolved in 20 mM sodium phosphate buffer, pH 7.5, (100 µL), before denaturation solution (2% SDS, 1 M 2-mercaptoethanol, 5 µL) was added and the samples were then heated at 100 °C for 5 min. NP-40 detergent (15% *v*/*v*, 5 µL) was then added, followed by N-glycanase (4 µL/20 mU) and then the samples were incubated at 37 °C for 3 h.

### 4.6. SDS PAGE and Detection of SLRP Fragments by Western Blotting

Aliquots of the chondroitinase ABC, keratanase-I, II or N-glycanase digested samples, containing tissue extracts from an equivalent wet weight of tissue (0.8 mg/lane), were mixed with 4× LDS PAGE application buffer (35 µL) and 10× reducing agent (15 µL) and re-dispersed in a total volume of 100 µL. The samples were heated at 70 °C for 30 min and then cooled, before 25 µL aliquots were electrophoresed under reducing conditions on 10% NuPAGE Bis-Tris gels at 200 V constant voltage for 50 min using NuPAGE MOPS SDS running buffer. The gels were electroblotted to nitrocellulose membranes (0.22 µm) using NuPAGE transfer buffer supplemented with 10% methanol at 30 V constant voltage for 1 h. SeeBlue-2 pre-stained protein molecular weight standards were also electrophoresed for molecular weight calibration and to assess the blotting transfer efficiency. The blots were initially blocked for 3 h with 5% BSA in 50 mM Tris-HCl 0.15 M NaCl pH 7.2 (TBS) then incubated with either PR-184 (0.5 µg/mL) or TsYG 11 (1/1000 diln) diluted in 2% BSA in TBS overnight at room temp. After a brief rinse in TBS, the goat anti rabbit IgG alkaline phosphatase conjugate diluted in TBS (1/5000 dilution) was added. After a further 1 h, the blots were washed in TBS (3 × 10 min) and the NBT/BCIP substrates were added into the alkaline phosphatase development buffer (0.1 M Tris-HCl pH 9.5 containing 5 mM MgCl_2_) for the detection of immune complexes. After development the blots were rinsed in milliQ distilled water before being dried. Western blots were repeated a minimum of three times and were also conducted omitting the primary antibody to check that no IgG species were present in the tissue extracts which cross-reacted with the conjugated secondary antibodies (false positives).

### 4.7. Cartilage Digestion with MMP-13, ADAMTS-4 and ADAMTS-5

Full-thickness articular cartilage (AC) was harvested from macroscopically normal regions of a femoral condyle of a 55 year-old-male cadaver (see Figure 2). The tissue was finely diced and 10 mg wet weight samples were aliquoted into eppendorf tubes. One cartilage sample was extracted directly with 4 M GuHCl buffered in 0.5 M sodium acetate, pH 5.8, containing 10 mM EDTA, 20 mM benzamidine and 50 mM 6-aminohexanoic acid (0.5 mL) with constant end-over-end mixing for 48 h at 4 °C. The extract was then recovered by centrifugation and the tissue residue discarded. In further tubes, diced AC was dispersed in: (i) a solution of 1 mM APMA and MMP-13 (50 µg/mL) in MMP digestion buffer (50 mM Tris HCl, 150 mM NaCl, 5 mM CaCl_2_, 1 mM ZnCl_2_, 0.01% Brij-35 pH 7.5 (0.2 mL)); (ii) 1 mM APMA in MMP digestion buffer; (iii) ADAMTS-4 or (iv) ADAMTS-5 (50 µg/mL) in the same MMP digestion buffer without APMA. After 24 h incubation at 37 °C, the tissue residues were spun down and the digestion buffer was collected. The tissue residues were extracted with 4 M GuHCl + proteinase inhibitors (0.5 mL) for 48 h at 4 °C, and then the extract was collected and the tissue residues were discarded. The 4 M GuHCl extracts and digestion buffer samples were dialyzed against milliQ distilled water then freeze dried. The samples were reconstituted in 50 mM Tris-acetate buffer pH 6.5 (0.2 mL) and the glycosaminoglycan (GAG) content of an aliquot was determined using the 1,9-dimethylmethylene blue metachromatic dye binding assay procedure. Chondroitinase ABC (0.01 U/10 μg GAG), keratanase-I (0.01 U/10 µg GAG) and keratanase-II (0.1 mU/10 µg GAG) were added to the samples and they were digested overnight at 37 °C. 4× LDS PAGE application buffer (80 µL) + 30 µL reducing agent were then added to the digested samples and they were heated on a block at 70 °C for 10 min. The samples were electrophoresed using pre-poured 10% Bis-Tris gels and transferred to nitrocellulose for analysis by Western blotting. Gel loading was 10 µg GAG/lane for BC-3, 20 µg GAG/lane for BC-14, and, for FMOD, the extract or digest supernatant from an equal wet weight of tissue (0.8 mg)/lane. Samples were pre-digested with chondroitinase ABC, and keratanase-I and II as indicated above.

### 4.8. Ovine Cartilage Explant Cultures

Full-depth articular cartilage explants were harvested from the trochlear groove of 6–12-month-old ovine knee joints and cultured at 37 °C, 5% (*v*/*v*) CO_2_ for 48 h in Dulbecco’s Modified Eagles Medium (DMEM; Sigma, Castle Hill, NSW, Australia), buffered with sodium bicarbonate 3.7 g/L (Fronine, Riverstone, NSW, Australia) and containing 10% (*v*/*v*) Fetal Calf Serum (FCS; Trace Biosciences Pty. Ltd., Castle Hill, NSW, Australia), 2 mM L-glutamine (ICN Biochemicals Inc., Aurora, OH, USA), and 50 µg/mL Gentamicin (Pharmacia Pty. Ltd., Bentley, WA, Australia). The explants were washed (3 × 5 min) in serum free DMEM and cultured individually in serum-free DMEM ± 5 ng/mL IL-1α plus 50 ng/mL oncostatin M (IL-1/OSM; PeproTech Rocky Hill, NJ 08553, United States) ± 300 nm PGE3162689 (synthesized at Procter and Gamble Pharmaceuticals, Cincinnati, Ohio, USA). As previously described, PGE3162689 at this concentration inhibited MMP-1, -2, -3, -7, -8, -9, -13 and -14 but not ADAMTSs in cartilage explant cultures [79]. The explants were cultured for 5 or 12 days with media changed at 5 and 12 days-(*n* = 8/treatments/time-point). At harvest, the explants (*n* = 6/group) were digested with papain, and the GAG and hydroxyproline content of the tissue digests and associated media were measured [79]. The remaining two randomly selected explants were pooled and extracted with 4 M GuHCl and the chondroitinase- and keratanase-digested extracts were analyzed by Western blotting with an antibody recognizing the C-terminus of bovine FMOD (PR-184) [76] as described above.

### 4.9. Histological Processing of Specimens

The human fetal feet were fixed in Histochoice for 18 h. Full-thickness slices (3-mm-thick) of adult femoral articular cartilage were fixed for 48 h in 10% neutral buffered formalin. The tissues were then dehydrated in graded ethanol solutions and finally with xylene and embedded in paraffin. The foot blocks underwent surface decalcification in 10 mM EDTA for 2 h prior to sectioning. Four micron vertical microtome sections were prepared and attached to positively charged microscope slides.

### 4.10. Toluidine Blue Staining of Cartilage Specimens

The cartilage sections (4 µm) were stained for 10 min with 0.04% (*w*/*v*) toluidine blue in 0.1 M sodium acetate buffer, pH 4.0, to visualize the anionic glycosaminoglycans followed by a 2-min counterstain in 0.1% (*w*/*v*) fast green FCF.

### 4.11. Immunolocalization of FMOD and MMP-13-Cleaved FMOD in Cartilage Tissue Sections

The tissue sections were rehydrated through graded ethanol washes and pre-digested with chondroitinase ABC (0.1 U) for 1 h, then with 0.3% H_2_O_2_ for 10 min to inactivate endogenous peroxidase activity and blocked with DAKO non-protein block. The primary antibodies to FMOD (PR-184, 0.5 µg/mL) or MMP-13-cleaved FMOD (TsYG11, 1/1000 dilution) in TBS containing 2% BSA were added to the sections and they were incubated overnight at 4 °C. The sections were then washed in TBS and biotinylated secondary antibody (mouse anti rabbit IgG, 5 µg/mL) was added for 3 h at room temperature. The color development was undertaken with avidin HRP conjugate using NovaRED peroxidase substrate for 20 min at room temperature and the slides were washed and mounted. The negative control sections were also run, omitting primary Ab or by substituting an irrelevant species specific primary antibody for the authentic one. Both yielded negative results.

### 4.12. Statistical Analyses

The hydroxyproline and sulfated GAG data were tested for significance using the Kruskal–Wallace equality-of-populations rank test in the first instance. If significance was found (*p* < 0.05), the samples were compared using the Wilcoxon rank-sum test. Within each parameter, the Benjamini–Hochberg post-hoc test was used to correct for false positives. All statistical analyses were performed using STATA 13 statistical software Survey Designs, College Station, Texas 77845, USA: StataCorp LP). Mean ± standard deviation data were plotted in histograms. Each analysis was based on six replicates and the experiment was repeated on at least two further samples. Data were considered significant when test data vs. control data groups displayed *p* < 0.05.

## 5. Conclusions

During cartilage development, remodeling, ageing and OA pathology, FMOD and LUM are both proteolytically processed to variable degrees. FMOD was susceptible to fragmentation by MMP-13, ADAMTS-4 and ADAMTS-5, the three major proteinases, which are also active during the development of OA but also with roles during cartilage development and remodeling. LUM, however, was relatively resistant to proteolysis by MMP-13 but was cleaved particularly by ADAMTS-4. While proteolytically susceptible to different exogenously added soluble enzymes, the in vitro stimulation of progressive chondrocyte-driven cartilage catabolism suggested that MMP-13 is the predominant enzyme responsible for cleavage of FMOD, which occurs after ADAMTS-driven aggrecanolysis. LUM and FMOD have roles in innate immune response and may modulate innate immune responses through pattern recognition receptors of relevance in cartilage homeostasis.

## Abbreviations

AC	articular cartilage
ADAMTS	A Disintegrin and Metalloproteinase with Thrombospondin motifs
AMPA	aminophenyl mercuric acetate
BGN	biglycan
BMI	body mass index
Brij-35®	Polyoxyethylene (23) lauryl ether
DAMPs	Damage-associated molecular pattern molecules
DCN	decorin
ECM	extracellular matrix
FMOD	fibromodulin
GuHCL	Guanidine hydrochloride
LRR	leucine-rich repeat
LUM	lumican
LuSDS	Lithium dodecyl sulphate
MOPS	3-(N-morpholino) propanesulfonic acid
OA	osteoarthritis
OSM	oncostatin M
MMP	matrix metalloprotease
SLRP	small leucine repeat proteoglycan
TLR	toll-like receptor
PRELP	proline/arginine-rich end leucine-rich repeat protein
PAGE	polyacrylamide gel electrophoresis

## References

[1] Wallace I.J., Worthington S., Felson D.T., Jurmain R.D., Wren K.T., Maijanen H., Woods R.J., Lieberman D.E. (2017). Knee osteoarthritis has doubled in prevalence since the mid-20th century. Proc. Natl. Acad. Sci. USA.

[2] Wluka A.E., Lombard C.B., Cicuttini F.M. (2013). Tackling obesity in knee osteoarthritis. Nat. Rev. Rheumatol..

[3] Cross M., Smith E., Hoy D., Nolte S., Ackerman I., Fransen M., Bridgett L., Williams S., Guillemin F., Hill C.L. (2014). The global burden of hip and knee osteoarthritis: Estimates from the global burden of disease 2010 study. Ann. Rheum. Dis..

[4] Felson D.T., Lawrence R.C., Dieppe P.A., Hirsch R., Helmick C.G., Jordan J.M., Kington R.S., Lane N.E., Nevitt M.C., Zhang Y. (2000). Osteoarthritis: New insights. Part 1: The disease and its risk factors. Ann. Intern. Med..

[5] Robinson W.H., Lepus C.M., Wang Q., Raghu H., Mao R., Lindstrom T.M., Sokolove J. (2016). Low-grade inflammation as a key mediator of the pathogenesis of osteoarthritis. Nat. Rev. Rheumatol.

[6] Chen A., Gupte C., Akhtar K., Smith P., Cobb J. (2012). The Global Economic Cost of Osteoarthritis: How the UK Compares. Arthritis.

[7] Troeberg L., Nagase H. (2012). Proteases involved in cartilage matrix degradation in osteoarthritis. Biochim. Biophys. Acta.

[8] Chen S., Oldberg A., Chakravarti S., Birk D.E. (2010). Fibromodulin regulates collagen fibrillogenesis during peripheral corneal development. Dev. Dyn..

[9] Chen S., Young M.F., Chakravarti S., Birk D.E. (2014). Interclass small leucine-rich repeat proteoglycan interactions regulate collagen fibrillogenesis and corneal stromal assembly. Matrix Biol.

[10] Jan A.T., Lee E.J., Choi I. (2016). Fibromodulin: A regulatory molecule maintaining cellular architecture for normal cellular function. Int. J. Biochem. Cell. Biol..

[11] Ni G.X., Li Z., Zhou Y.Z. (2014). The role of small leucine-rich proteoglycans in osteoarthritis pathogenesis. Osteoarthritis Cartilage.

[12] Iozzo R.V., Schaefer L. (2015). Proteoglycan form and function: A comprehensive nomenclature of proteoglycans. Matrix Biol.

[13] Ezura Y., Chakravarti S., Oldberg A., Chervoneva I., Birk D.E. (2000). Differential expression of lumican and fibromodulin regulate collagen fibrillogenesis in developing mouse tendons. J. Cell. Biol..

[14] Kalamajski S., Oldberg A. (2009). Homologous sequence in lumican and fibromodulin leucine-rich repeat 5-7 competes for collagen binding. J. Biol. Chem..

[15] Svensson L., Narlid I., Oldberg A. (2000). Fibromodulin and lumican bind to the same region on collagen type I fibrils. FEBS Lett..

[16] Gill M.R., Oldberg A., Reinholt F.P. (2002). Fibromodulin-null murine knee joints display increased incidences of osteoarthritis and alterations in tissue biochemistry. Osteoarthritis Cartilage.

[17] Svensson L., Aszodi A., Reinholt F.P., Fassler R., Heinegard D., Oldberg A. (1999). Fibromodulin-null mice have abnormal collagen fibrils, tissue organization, and altered lumican deposition in tendon. J. Biol. Chem..

[18] Onnerfjord P., Heathfield T.F., Heinegard D. (2004). Identification of tyrosine sulfation in extracellular leucine-rich repeat proteins using mass spectrometry. J. Biol. Chem..

[19] Tillgren V., Onnerfjord P., Haglund L., Heinegard D. (2009). The tyrosine sulfate-rich domains of the LRR proteins fibromodulin and osteoadherin bind motifs of basic clusters in a variety of heparin-binding proteins, including bioactive factors. J. Biol. Chem..

[20] Tillgren V., Morgelin M., Onnerfjord P., Kalamajski S., Aspberg A. (2016). The Tyrosine Sulfate Domain of Fibromodulin Binds Collagen and Enhances Fibril Formation. J. Biol. Chem..

[21] Hildebrand A., Romaris M., Rasmussen L.M., Heinegard D., Twardzik D.R., Border W.A., Ruoslahti E. (1994). Interaction of the small interstitial proteoglycans biglycan, decorin and fibromodulin with transforming growth factor beta. Biochem. J..

[22] Soo C., Hu F.Y., Zhang X., Wang Y., Beanes S.R., Lorenz H.P., Hedrick M.H., Mackool R.J., Plaas A., Kim S.J. (2000). Differential expression of fibromodulin, a transforming growth factor-beta modulator, in fetal skin development and scarless repair. Am. J. Pathol..

[23] Sjoberg A., Onnerfjord P., Morgelin M., Heinegard D., Blom A.M. (2005). The extracellular matrix and inflammation: Fibromodulin activates the classical pathway of complement by directly binding C1q. J. Biol. Chem..

[24] Brezillon S., Pietraszek K., Maquart F.X., Wegrowski Y. (2013). Lumican effects in the control of tumour progression and their links with metalloproteinases and integrins. FEBS J..

[25] Niewiarowska J., Brezillon S., Sacewicz-Hofman I., Bednarek R., Maquart F.X., Malinowski M., Wiktorska M., Wegrowski Y., Cierniewski C.S. (2011). Lumican inhibits angiogenesis by interfering with alpha2beta1 receptor activity and downregulating MMP-14 expression. Thromb. Res..

[26] Stasiak M., Boncela J., Perreau C., Karamanou K., Chatron-Colliet A., Proult I., Przygodzka P., Chakravarti S., Maquart F.X., Kowalska M.A. (2016). Lumican Inhibits SNAIL-Induced Melanoma Cell Migration Specifically by Blocking MMP-14 Activity. PLoS ONE.

[27] Geng Y., McQuillan D., Roughley P.J. (2006). SLRP interaction can protect collagen fibrils from cleavage by collagenases. Matrix Biol..

[28] Cs-Szabo G., Melching L.I., Roughley P.J., Glant T.T. (1997). Changes in messenger RNA and protein levels of proteoglycans and link protein in human osteoarthritic cartilage samples. Arthritis Rheum..

[29] Melrose J., Fuller E.S., Roughley P.J., Smith M.M., Kerr B., Hughes C.E., Caterson B., Little C.B. (2008). Fragmentation of decorin, biglycan, lumican and keratocan is elevated in degenerate human meniscus, knee and hip articular cartilages compared with age-matched macroscopically normal and control tissues. Arthritis Res. Ther..

[30] Zhen E.Y., Brittain I.J., Laska D.A., Mitchell P.G., Sumer E.U., Karsdal M.A., Duffin K.L. (2008). Characterization of metalloprotease cleavage products of human articular cartilage. Arthritis Rheum..

[31] Danfelter M., Onnerfjord P., Heinegard D. (2007). Fragmentation of proteins in cartilage treated with interleukin-1: Specific cleavage of type IX collagen by matrix metalloproteinase 13 releases the NC4 domain. J. Biol. Chem..

[32] Little C.B., Barai A., Burkhardt D., Smith S.M., Fosang A.J., Werb Z., Shah M., Thompson E.W. (2009). Matrix metalloproteinase 13-deficient mice are resistant to osteoarthritic cartilage erosion but not chondrocyte hypertrophy or osteophyte development. Arthritis Rheum.

[33] Kashiwagi M., Enghild J.J., Gendron C., Hughes C., Caterson B., Itoh Y., Nagase H. (2004). Altered proteolytic activities of ADAMTS-4 expressed by C-terminal processing. J. Biol. Chem..

[34] Flannery C.R. (2006). MMPs and ADAMTSs: Functional studies. Front. Biosci..

[35] Flannery C.R., Zeng W., Corcoran C., Collins-Racie L.A., Chockalingam P.S., Hebert T., Mackie S.A., McDonagh T., Crawford T.K., Tomkinson K.N. (2002). Autocatalytic cleavage of ADAMTS-4 (Aggrecanase-1) reveals multiple glycosaminoglycan-binding sites. J. Biol. Chem..

[36] Jackson M.T., Moradi B., Smith M.M., Jackson C.J., Little C.B. (2014). Activation of matrix metalloproteinases 2, 9, and 13 by activated protein C in human osteoarthritic cartilage chondrocytes. Arthritis Rheum..

[37] Jackson M.T., Moradi B., Zaki S., Smith M.M., McCracken S., Smith S.M., Jackson C.J., Little C.B. (2014). Depletion of protease-activated receptor 2 but not protease-activated receptor 1 may confer protection against osteoarthritis in mice through extracartilaginous mechanisms. Arthritis Rheum..

[38] Jackson M.T., Smith M.M., Smith S.M., Jackson C.J., Xue M., Little C.B. (2009). Activation of cartilage matrix metalloproteinases by activated protein C. Arthritis Rheum..

[39] D’Angelo M., Yan Z., Nooreyazdan M., Pacifici M., Sarment D.S., Billings P.C., Leboy P.S. (2000). MMP-13 is induced during chondrocyte hypertrophy. J. Cell. Biochem..

[40] Favero M., Belluzzi E., Trisolino G., Goldring M.B., Goldring S.R., Cigolotti A., Pozzuoli A., Ruggieri P., Ramonda R., Grigolo B. (2018). Inflammatory molecules produced by meniscus and synovium in early and end-stage osteoarthritis: A coculture study. J. Cell Physiol..

[41] Favero M., Ramonda R., Goldring M.B., Goldring S.R., Punzi L. (2015). Early knee osteoarthritis. RMD Open.

[42] Belluzzi E., El Hadi H., Granzotto M., Rossato M., Ramonda R., Macchi V., De Caro R., Vettor R., Favero M. (2017). Systemic and Local Adipose Tissue in Knee Osteoarthritis. J. Cell. Physiol..

[43] Chang J., Liao Z., Lu M., Meng T., Han W., Ding C. (2018). Systemic and local adipose tissue in knee osteoarthritis. Osteoarthritis Cartilage.

[44] Favero M., El-Hadi H., Belluzzi E., Granzotto M., Porzionato A., Sarasin G., Rambaldo A., Iacobellis C., Cigolotti A., Fontanella C.G. (2017). Infrapatellar fat pad features in osteoarthritis: A histopathological and molecular study. Rheumatology (Oxford).

[45] Goldring S.R. (2012). Alterations in periarticular bone and cross talk between subchondral bone and articular cartilage in osteoarthritis. Ther. Adv. Musculoskelet. Dis..

[46] Richter M., Trzeciak T., Owecki M., Pucher A., Kaczmarczyk J. (2015). The role of adipocytokines in the pathogenesis of knee joint osteoarthritis. Int. Orthop..

[47] Scanzello C.R., Goldring S.R. (2012). The role of synovitis in osteoarthritis pathogenesis. Bone.

[48] Fuller E., Little C.B., Melrose J. (2016). Interleukin-1alpha induces focal degradation of biglycan and tissue degeneration in an in-vitro ovine meniscal model. Exp. Mol. Pathol..

[49] Fuller E.S., Smith M.M., Little C.B., Melrose J. (2012). Zonal differences in meniscus matrix turnover and cytokine response. Osteoarthritis Cartilage.

[50] Melrose J., Fuller E.S., Little C.B. (2017). The biology of meniscal pathology in osteoarthritis and its contribution to joint disease: Beyond simple mechanics. Connect. Tissue Res..

[51] Madry H., Kon E., Condello V., Peretti G.M., Steinwachs M., Seil R., Berruto M., Engebretsen L., Filardo G., Angele P. (2016). Early osteoarthritis of the knee. Knee Surg. Sports Traumatol. Arthrosc..

[52] Malempati C., Jacobs C.A., Lattermann C. (2017). The Early Osteoarthritic Knee: Implications for Cartilage Repair. Clin. Sports Med..

[53] Matsubara H., Okazaki K., Takayama Y., Osaki K., Matsuo Y., Honda H., Iwamoto Y. (2015). Detection of early cartilage deterioration associated with meniscal tear using T1rho mapping magnetic resonance imaging. BMC Musculoskelet. Disord..

[54] Williams A., Winalski C.S., Chu C.R. (2017). Early articular cartilage MRI T2 changes after anterior cruciate ligament reconstruction correlate with later changes in T2 and cartilage thickness. J. Orthop. Res..

[55] Liu-Bryan R. (2013). Synovium and the innate inflammatory network in osteoarthritis progression. Curr. Rheum. Rep..

[56] Merline R., Schaefer R.M., Schaefer L. (2009). The matricellular functions of small leucine-rich proteoglycans (SLRPs). J. Cell. Commun. Signal..

[57] Rosenberg J.H., Rai V., Dilisio M.F., Agrawal D.K. (2017). Damage-associated molecular patterns in the pathogenesis of osteoarthritis: Potentially novel therapeutic targets. Mol. Cell. Biochem..

[58] Miller R.E., Belmadani A., Ishihara S., Tran P.B., Ren D., Miller R.J., Malfait A.M. (2015). Damage-associated molecular patterns generated in osteoarthritis directly excite murine nociceptive neurons through Toll-like receptor 4. Arthritis Rheum..

[59] Ricard-Blum S., Ballut L. (2011). Matricryptins derived from collagens and proteoglycans. Front. Biosci. (Landmark Ed.).

[60] Moreth K., Iozzo R.V., Schaefer L. (2012). Small leucine-rich proteoglycans orchestrate receptor crosstalk during inflammation. Cell Cycle.

[61] Nastase M.V., Janicova A., Roedig H., Hsieh L.T., Wygrecka M., Schaefer L. (2018). Small Leucine-Rich Proteoglycans in Renal Inflammation: Two Sides of the Coin. J. Histochem. Cytochem..

[62] Schaefer L. (2014). Complexity of danger: The diverse nature of damage-associated molecular patterns. J. Biol. Chem..

[63] Stanton H., Melrose J., Little C.B., Fosang A.J. (2011). Proteoglycan degradation by the ADAMTS family of proteinases. Biochim. Biophys. Acta.

[64] Pietraszek K., Chatron-Colliet A., Brezillon S., Perreau C., Jakubiak-Augustyn A., Krotkiewski H., Maquart F.X., Wegrowski Y. (2014). Lumican: A new inhibitor of matrix metalloproteinase-14 activity. FEBS Lett..

[65] Pietraszek K., Brezillon S., Perreau C., Malicka-Blaszkiewicz M., Maquart F.X., Wegrowski Y. (2013). Lumican -derived peptides inhibit melanoma cell growth and migration. PLoS ONE.

[66] Li Y., Aoki T., Mori Y., Ahmad M., Miyamori H., Takino T., Sato H. (2004). Cleavage of lumican by membrane-type matrix metalloproteinase-1 abrogates this proteoglycan-mediated suppression of tumor cell colony formation in soft agar. Cancer Res..

[67] Heathfield T.F., Onnerfjord P., Dahlberg L., Heinegard D. (2004). Cleavage of fibromodulin in cartilage explants involves removal of the N-terminal tyrosine sulfate-rich region by proteolysis at a site that is sensitive to matrix metalloproteinase-13. J. Biol. Chem..

[68] Young A.A., Smith M.M., Smith S.M., Cake M.A., Ghosh P., Read R.A., Melrose J., Sonnabend D.H., Roughley P.J., Little C.B. (2005). Regional assessment of articular cartilage gene expression and small proteoglycan metabolism in an animal model of osteoarthritis. Arthritis Res. Ther..

[69] Hedlund H., Mengarelli-Widholm S., Heinegard D., Reinholt F.P., Svensson O. (1994). Fibromodulin distribution and association with collagen. Matrix Biol..

[70] Cs-Szabo G., Roughley P.J., Plaas A.H., Glant T.T. (1995). Large and small proteoglycans of osteoarthritic and rheumatoid articular cartilage. Arthritis Rheum..

[71] Roughley P.J., White R.J., Cs-Szabo G., Mort J.S. (1996). Changes with age in the structure of fibromodulin in human articular cartilage. Osteoarthritis Cartilage.

[72] Melrose J., Fuller E.S., Roughley P.J., Smith M.M., Kerr B., Hughes C.E., Caterson B., Little C.B. (2008). Fragmentation of Decorin, Biglycan, Lumican and Keratocan is elevated in Degenerate Human Meniscus, Knee and Hip Articular Cartilages compared to Age-matched Macroscopically Normal and Control Tissues. Arthritis Res. Ther..

[73] Sztrolovics R., White R.J., Poole A.R., Mort J.S., Roughley P.J. (1999). Resistance of small leucine-rich repeat proteoglycans to proteolytic degradation during interleukin-1-stimulated cartilage catabolism. Biochem. J..

[74] Gendron C., Kashiwagi M., Lim N.H., Enghild J.J., Thogersen I.B., Hughes C., Caterson B., Nagase H. (2007). Proteolytic activities of human ADAMTS-5: Comparative studies with ADAMTS-4. J. Biol. Chem..

[75] Fushimi K., Troeberg L., Nakamura H., Lim N.H., Nagase H. (2008). Functional differences of the catalytic and non-catalytic domains in human ADAMTS-4 and ADAMTS-5 in aggrecanolytic activity. J. Biol. Chem..

[76] Sztrolovics R., Alini M., Mort J.S., Roughley P.J. (1999). Age-related changes in fibromodulin and lumican in human intervertebral discs. Spine.

[77] Ge G., Seo N.S., Liang X., Hopkins D.R., Hook M., Greenspan D.S. (2004). Bone morphogenetic protein-1/tolloid-related metalloproteinases process osteoglycin and enhance its ability to regulate collagen fibrillogenesis. J. Biol. Chem..

[78] Melrose J., Smith S.M., Fuller E.S., Young A.A., Roughley P.J., Dart A., Little C.B. (2007). Biglycan and fibromodulin fragmentation correlates with temporal and spatial annular remodelling in experimentally injured ovine intervertebral discs. Eur. Spine J..

[79] Little C.B., Hughes C.E., Curtis C.L., Janusz M.J., Bohne R., Wang-Weigand S., Taiwo Y.O., Mitchell P.G., Otterness I.G., Flannery C.R. (2002). Matrix metalloproteinases are involved in C-terminal and interglobular domain processing of cartilage aggrecan in late stage cartilage degradation. Matrix Biol..

